# Seasons, weather, and device-measured movement behaviors: a scoping review from 2006 to 2020

**DOI:** 10.1186/s12966-021-01091-1

**Published:** 2021-02-04

**Authors:** Taylor B. Turrisi, Kelsey M. Bittel, Ashley B. West, Sarah Hojjatinia, Sahar Hojjatinia, Scherezade K. Mama, Constantino M. Lagoa, David E. Conroy

**Affiliations:** 1 Department of Kinesiology, The Pennsylvania State University, University Park, PA 16802 USA; 2 Advanced Safety & User Experience, Aptiv, Troy, MI USA; 3 Department of Electrical Engineering & Computer Science, The Pennsylvania State University, University Park, PA USA; 4 Department of Health Disparities Research, The University of Texas MD Anderson Cancer Center, Houston, TX USA; 5 Department of Preventive Medicine, Northwestern University, Chicago, IL USA

**Keywords:** Environment, Seasons, Meteorological concepts, Rain, Sunlight, Temperature, Wind, Exercise, Screen time

## Abstract

**Background:**

This scoping review summarized research on (a) seasonal differences in physical activity and sedentary behavior, and (b) specific weather indices associated with those behaviors.

**Methods:**

PubMed, CINAHL, and SPORTDiscus were searched to identify relevant studies. After identifying and screening 1459 articles, data were extracted from 110 articles with 118,189 participants from 30 countries (almost exclusively high-income countries) on five continents.

**Results:**

Both physical activity volume and moderate-to-vigorous physical activity (MVPA) were greater in summer than winter. Sedentary behavior was greater in winter than either spring or summer, and insufficient evidence existed to draw conclusions about seasonal differences in light physical activity. Physical activity volume and MVPA duration were positively associated with both the photoperiod and temperature, and negatively associated with precipitation. Sedentary behavior was negatively associated with photoperiod and positively associated with precipitation. Insufficient evidence existed to draw conclusions about light physical activity and specific weather indices. Many weather indices have been neglected in this literature (e.g., air quality, barometric pressure, cloud coverage, humidity, snow, visibility, windchill).

**Conclusions:**

The natural environment can influence health by facilitating or inhibiting physical activity. Behavioral interventions should be sensitive to potential weather impacts. Extreme weather conditions brought about by climate change may compromise health-enhancing physical activity in the short term and, over longer periods of time, stimulate human migration in search of more suitable environmental niches.

**Supplementary Information:**

The online version contains supplementary material available at 10.1186/s12966-021-01091-1.

The global prevalence of insufficient physical activity is approximately 28% but exceeds 40% in some regions [1]. Physical activity promotion efforts have targeted determinants at multiple levels of the socio-ecological model, including the person (e.g., motivation), social environment (e.g., family, peers), and built environment (e.g., access to equipment, neighborhood walkability) [2–4]. The *natural environment* includes a number of factors that influence physical activity, including seasons and weather. Weather is often cited as a perceived barrier to participation in movement behaviors [5–9]. Although weather conditions are not acutely modifiable, they are important to understand because of their ability to alter opportunities for physical activity and moderate the effectiveness of interventions targeting determinants at other levels.

Two seminal reviews of research on seasonality, weather, and physical activity across the lifespan were published over a decade ago [10, 11]. Physical activity was typically greatest during spring and summer and lowest during winter, but regions with more extreme weather conditions sometimes yielded different conclusions. For example, a study conducted in Galveston, Texas, where the average temperature during summer months is over 28 °C (82 °F), revealed lower levels of physical activity in the summer than in winter [12]. However, the general pattern of seasonal trends demonstrated that people accumulated greater levels of physical activity in warmer and more arid conditions. Weather conditions were also found to have differing impacts on physical activity between sub-groups in the population [10, 13, 14]. For instance, physical activity was not associated with wind gusts for most individuals, but individuals with lower body mass were less active in the presence of stronger wind gusts than individuals with higher body mass [15].

The social context for research on weather and physical activity changed in three significant ways around the time of the seminal Tucker and Gilliland review [10]. First, public interest in weather and health increased following the 2007 Nobel Peace Prize that was awarded jointly to the Intergovernmental Panel on Climate Change and former US Vice President Al Gore [16]. As Earth’s surface temperature rises, extreme weather conditions will increase air pollution and ultraviolet radiation exposure, increasing risk for cardiovascular and lung diseases as well as cancer [17]. Second, the mobile and wearable technology industries experienced major disruptions in 2007 due to the launch of the Apple iPhone and the founding of Fitbit [18, 19]. The widespread adoption of mobile technologies such as smartphones and wearable activity monitors enabled researchers to monitor both physical activity and location-specific weather indices in real-time. Third, sedentary behavior – waking activity conducted in a seated or reclined posture involving low energy expenditure – has emerged as a distinct behavior that has important health consequences independent of physical activity levels [20, 21].

Despite summarizing over 60 studies with over 300,000 participants from approximately 18 countries, these seminal reviews possess several limitations. First, although they both examined seasonal differences in physical activity, most findings at the time focused on a relatively narrow range of specific weather indices with temperature and precipitation being most common. Understanding associations between additional weather indices and physical activity could both explain seasonal differences and facilitate the development of just-in-time interventions that leverage information from short-term weather forecasts. Second, weather and physical activity data were aggregated into person-level summary measures, yet both weather and physical activity are dynamic. Understanding the timing of and changes in weather conditions is essential because current weather conditions are likely to have a more immediate influence on physical activity than average weather conditions. Third, both reviews focused on a general physical activity outcome. Physical activity can be quantified as total volume (to represent energy expended) or the duration of activities completed at specific intensities (to represent time allocated to different effort levels). Sedentary behavior may also be impacted as weather conditions alter people’s activity choices. Additional reviews published since these seminal reviews have been limited by incomplete search strategies and a focus on narrow segments of the population [14, 22]. In light of these limitations and the broader context described earlier (increasing public interest in climate, advances in mobile technology, the emergence of sedentary behavior), an updated review of weather and movement behavior, including both physical activity and sedentary behavior, would be a valuable contribution.

A scoping review was conducted to examine associations between device-based measures of physical activity, sedentary behavior and a broad array of weather-related phenomena at different levels of specificity, ranging from seasons (e.g., spring, summer, fall, winter) to specific weather indices (e.g., humidity, precipitation, temperature). A scoping review was selected over a systematic review or meta-analysis based on the breadth of weather indices available and the need to both analyze the available evidence and identify existing knowledge gaps [23].

## Methods

### Search strategy

PubMed, the Cumulative Index of Nursing and Allied Health Literature (CINAHL), and SPORTDiscus electronic databases were searched from January 1, 2006 to October 31, 2020. This date range was chosen to capture all research since the end of the search period used by Tucker and Gilliland [10]. Three databases were selected based on their likelihood of including both environmental and health behavior data. Three main subject categories were included in our searches: movement-related behavior, movement-related behavior measurement (i.e., intensity, volume), and weather. Movement-related behavior search terms related to physical activity or sedentary behavior were combined with “or” statements. These terms mirrored the search terms used to compile the literature for the 2018 Physical Activity Guidelines Advisory Committee [24]. Measurement search terms related to technologies commonly used to measure physical activity were combined with “or” statements (e.g., accelerometer or pedometer). Weather search terms related to seasonality or specific weather indices were combined with “or” statements (e.g., seasons or temperature or humidity or precipitation). The search strategies for each database used are available in Appendices 1, 2, and 3. The searches were restricted to articles that (a) were written in English, (b) examined human subjects only, and (c) included empirical studies only (i.e., no review papers). This search strategy was constructed in consultation with a trained reference librarian and search specialist at The Pennsylvania State University. The review protocol was not preregistered.

### Selection process

Articles were included if (a) physical activity or sedentary behavior was an outcome variable of interest, (b) physical activity data were collected using device-based measurements (e.g., accelerometer, pedometer), and (c) results involved associations or differences between either movement-related behavior and either seasonality or weather indices. Articles were excluded if (a) studies were not published in English, (b) samples included non-human subjects, (c) results were limited to prevalence rates or other descriptive data, or (d) physical activity data were collected using self-report measures exclusively.

**Fig. 1:**
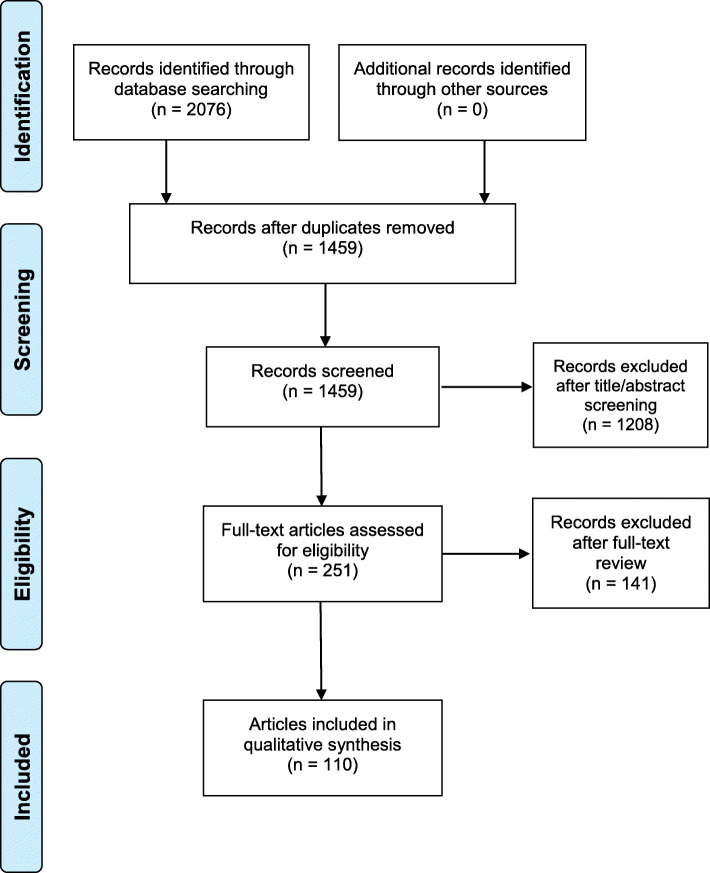
Process of Identification and Screening for Included Articles

Titles and abstracts were independently reviewed in a blinded manner by coders trained by the first author using the eligibility criteria described above. The first author exported citations for each article found in the PubMed, CINAHL, and SPORTDiscus searches, and uploaded these citations into Rayyan. Rayyan is a web application that is used to facilitate collaborative screening of titles and abstracts for reviews [25, 26]. Each coder accessed their assigned articles via the Rayyan web interface, reviewed titles and abstracts, and recorded decisions to include or exclude each article. Figure 1 summarizes study selection [27].

### Data extraction

Articles containing studies that met inclusion criteria during full-text review were advanced for data extraction. Prior to data extraction, the first author and four trained coders used a standardized coding guide to code three identical papers that were eligible for data extraction. The coding guide was developed to ensure that sufficient descriptive information was collected to properly characterize associations of interest. The coders then met to compare codes and discuss disagreements to promote consistency for final extraction. After this calibration exercise, each coder independently extracted data from the remaining articles using the standardized coding guide. Prior to analysis, the first author reviewed each article to ensure that the necessary data was extracted in a manner consistent with the coding guide.

A copy of the coding guide is available in supplementary online files. Extracted sample characteristics included age, sex, education, race, and the country where the data were collected. If countries included multiple diverse climate zones, the region of the country was also collected. Physical activity, sedentary behavior, weather indices (temperature, precipitation, wind speed, photoperiod, snow, cloud coverage, humidity, visibility, barometric pressure, windchill, and air quality; see Appendix 4 for definitions), and seasonality were all characterized by assessment timeframe and method of measurement. Research designs were classified as cross-sectional or longitudinal. The following statistics were extracted when available: t-scores, F-scores, correlation coefficients, β coefficients, *p*-values, effect sizes, and odds ratios. Significance thresholds were kept consistent with the author’s prespecified level of significance, and all results were coded having a positive or negative association, or failing to reach significance.

### Evidence grading

All of the available evidence from independent samples was observational so all studies were deemed to have a high risk of bias. Instead of rating study-level bias, evidence was graded based on the quantity and consistency of findings when five of more studies were available for a specific comparison or association. A “strong” grade was assigned when a conclusion was based on highly consistent findings related to the direction of a difference or association (or moderately consistent findings when a large number of studies was available). A “moderate” grade was assigned when a conclusion was based on mixed findings but the preponderance of evidence pointed to a consistent direction of a difference or association. A “limited” grade was assigned when a conclusion could not be drawn because of equivocal evidence related to the direction of the difference or association. When fewer than five studies were available, we noted that a grade was not assignable. This grading system was drawn from criteria used by the 2018 Physical Activity Guidelines Advisory Committee and adapted to match the state of this literature [24].

## Results

A total of 1459 unique articles were identified during the initial search. Following title and abstract screening, 1208 articles were excluded. Full-text review of the remaining 251 articles led to an additional 141 exclusions. A total of 110 articles reporting 144 studies of independent samples were identified as eligible for inclusion in this review. Among those 110 articles, 26 reported two independent samples, one reported three independent samples, and two reported four independent samples to examine physical activity behaviors between sex, age group, or region, or examined both seasonality and weather [28–44].

**Fig. 2:**
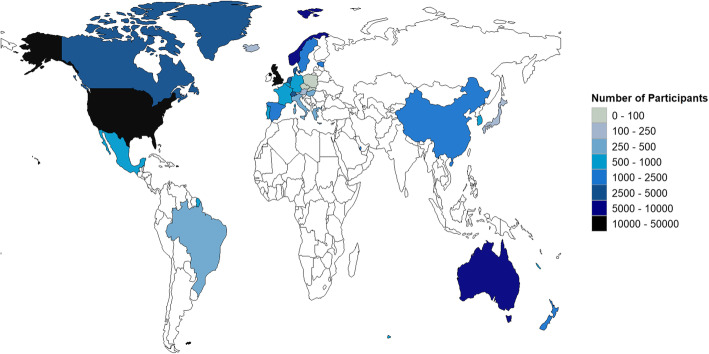
Geographic distribution of participants included in this review

Table 1 summarizes participant characteristics and study designs for all included articles. In total, articles included 118,189 participants (62.0% female participants, median *N* = 272, IQR = 85–722) from 30 unique countries on five continents. Figure 2 summarizes the sampling density across the globe. Most data represented western countries such as the United Kingdom, United States, Norway, Australia, Denmark, and Canada. Studies were primarily conducted in countries that the World Bank currently (July 2020) classifies as high income (27/30, 90.0%) and three were upper-middle income (3/30, 10.0%) [45]. None were from low- or middle-income countries.

**Table 1:** Sample and design characteristics of eligible articles

Reference	Country	N	Female (%)	Age Group	Age (***M*** ± ***SD*** [years])	Race	Monitoring Period	Design	Environmental Measure
Aadland et al. (2018)	Norway	465	51.6	Youth	10.9 ± 0.3	NA	7 days	Longitudinal	Season
Aebi et al. (2020)	Switzerland	1314	48.7	Adults	67.9 ± 7.9	NA	8 days	Cross-sectional	Season
Aibar Solana et al. (2015)	France, Spain	646	58.8	Youth	14.3 ± 0.7	NA	7 days	Longitudinal	Weather
Akande et al. (2019)	Canada	272	43.8	Adults	34.9 ± 12.6	Inuit: 74.6%; Other: 25.4%	7 days	Longitudinal	
Albrecht et al. (2020)	Germany	577	52.0	Older adults	Range: 65–75	NA	7 days	Longitudinal	
Al-Mohannadi et al. (2016)	Qatar	2088	33.4	Adults	41.6 ± 10.7	Eastern Mediterranean: 52.3%; South East Asian: 30.7%; Western Pacific:8.0%; African: 3.4%; North/South American: 3.1%; European: 2.5%	24 months	Longitudinal	
Arnardottir et al. (2017)	Iceland	138	60.1	Older adults	80.3 ± 4.9	NA	7 days	Cross-sectional	Season
Aspvik et al. (2018)	Norway	1219	51.2	Older adults	72.4 ± 2.1	NA	7 days	Longitudinal	Weather
Atkin et al. (2016)	United Kingdom, Wales, Scotland, Northern Ireland	704	52.6	Youth	7.6 ± 0.3	Caucasian: 92.9%; Other: 7.1%	7 days	Longitudinal	Weather
Badland et al. (2011)	Australia	1754	59.3	Adults	39.9 ± 11.8	NA	7 days	Longitudinal	Season, Weather
Balish et al. (2017)	Canada	190	45.3	Adults	NA	NA	7 days	Longitudinal	Season, Weather
Barkley and Herrmann (2017)	United States	16	NA	Older adults	NA	NA	7 days	Longitudinal	Season
Beighle et al. (2013)	United States	321	52.0	Youth	9.1 ± 1.5	NA	7 days	Longitudinal	Season
Bejarano et al. (2019)	United States	26	42.3	Youth	16 ± 1.6	Caucasian: 69.2%; Native American: 15.4%; Hispanic: 11.5%; Asian: 3.8%	20 days	Longitudinal	Weather
Boutou et al. (2019)	United Kingdom, Belgium, Greece, Netherlands	157	75.8	Adults	67.2 ± 7.8	NA	7 days	Longitudinal	Weather
Brandon et al. (2009)	Canada	48	75.0	Older adults	77.4 ± 4.7	NA	7 days	Longitudinal	Weather
Bremer et al. (2019)	Canada	110	51.8	Youth	10.2 ± 1.7	NA	7 days	Longitudinal	Weather
Bringolf-Isler et al. (2009)	Switzerland	164	52.0	Youth	Range: 6–14	NA	7 days	Longitudinal	Season
Brychta et al. (2016)	Iceland	70	64.3	Older adults	79.5 ± 4.8	NA	7 days	Cross-sectional	Weather
Buchowski et al. (2009)	United States	57	100	Adults	36.5 ± 9.2	NA	7 days	Longitudinal	Season
Button et al. (2020)	Canada	90	61.1	Youth	10.6 ± 1.4	White: 56.7%; Indigenous & Visible Minority: 43.3%	8 days	Longitudinal	Weather
Carr et al. (2016)	United States	132	100	Adults	41.6 ± 10.1	Hispanic/Latino: 100%	12 months	Longitudinal	Season
Cepeda et al. (2018)	Netherlands	1166	44.4	Adults & older adults	Middle aged: Median = 59.1 [IQR = 56.1–62.4]; Young elderly: 71.3 [67.3–72.8]; Old elderly: 78.9 [77.0–81.4]	NA	7 days	Cross-sectional	Season
Chang et al. (2020)	China	53	49.1	Youth	4.9 ± 0.2	NA	7 days	Longitudinal	Season
Clemes et al. (2011)	United Kingdom	96	53.2	Adults	40.7 ± 12.5	NA	4 weeks	Longitudinal	Season
Collings et al. (2020)	United Kingdom	342	48.8	Youth	3.4 ± 0.8	White: 40.9%; South Asian: 59.1%	6 days	Longitudinal	Season
Colom et al. (2019)	Spain	218	51.0	Adults	Range: 55–75	NA	9 days	Cross-sectional	Weather
Cooper et al. (2010)	United Kingdom	1010	53.3	Youth	11.0 ± 0.4	NA	4 days	Cross-sectional	Season
Cradock et al. (2009)	United States	152	41.0	Youth	13.7 ± 0.7	White: 57.0%; Black/African-American: 10.0%; Hispanic: 13.0%; Asian: 12.0%; Other Race/Ethnicity: 9.0%	4 days	Cross-sectional	Weather
Crowley et al. (2016)	United States	510	55.0	Adults	43.5 ± 9.2	NA	12 months	Longitudinal	Season
Cullen et al. (2017)	United States	342	100	Youth	Range: 8–10	African-American: 100%	7 days	Cross-sectional	Weather
Davis et al. (2011)	United Kingdom	230	49.1	Older adults	78.1 ± 5.8		7 days	Longitudinal	Season, Weather
de Vries et al. (2019)	Netherlands	10	50.0	Youth	12.5	NA	14 days	Longitudinal	Season, Weather
Declòs-Alió et al. (2019)	Spain	227	56.0	Older adults	NA	NA	7 days	Longitudinal	Season
Deng and Fredriksen (2018)	Norway	2123	49.6	Youth	9 ± 1.5	NA	7 days	Cross-sectional	Season
Dias et al. (2019)	Switzerland, Belgium, United Kingdom, United States	1052	49.8	Youth	3.7 ± 0.4	NA	3 days	Longitudinal	Season, Weather
Diaz et al. (2016)	United States	2096	54.2	Adults	Range: 45–75	Black: 31.6%	7 days	Cross-sectional	Season
Dill et al. (2014)	United States	353	NA	Adults	NA		3 days	Longitudinal	Weather
Duncan et al. (2008)	New Zealand	1115	51.9	Youth	Range: 5–16	Caucasian: 49.2%; Polynesian: 30.0%; Asian: 16.5%; Other: 4.3%	5 days	Cross-sectional	Season
Edwards et al. (2015)	United States	372	48.0	Youth	3.4 ± 0.3	Caucasian: 88.0% African-American: 22.0%	3 days	Longitudinal	Season
Feinglass et al. (2011)	United States	241	74.7	Adults	< 50 years: (26.1%); 50–65 years: (39.3%); 66–75 years: (22.2%); > 75 years: (12.5%)	Caucasian/ Other: 75.1%; African-American: 17.8%; Hispanic/ Latino: 7.1%	7 days	Longitudinal	Weather
Goodman et al. (2012)	United Kingdom	325	52.3	Youth	Range: 8–11	NA	4 days	Longitudinal	Weather
Goodman et al. (2014)	Australia, Brazil, Denmark, England, Estonia, Spain, Norway, Switzerland, United States	23,188	62.0	Youth	Range: 5–16	NA	7 days	Longitudinal	Weather
Gracia-Marco et al. (2013)	Sweden, Greece, Italy, Spain, Hungary, Belgium, France, Germany, Austria	2173	54.0	Youth	15 ± 1.2	NA	7 days	Longitudinal	Season
Griew et al. (2010)	United Kingdom	1307	51.8	Youth	Range: 10–11	NA	5 days	Longitudinal	Weather
Hagströmer et al. (2014)	Sweden	1172	54.0	Adults	45 ± 15	NA	7 days	Longitudinal	Season
Hamilton et al. (2009)	United Kingdom	96	51.1	Adults	41.0 ± 12.3	NA	4 weeks	Longitudinal	Weather
Harrison et al. (2011)	United Kingdom	1794	55.0	Youth	Range: 9–10	NA	7 days	Longitudinal	Weather
Harrison et al. (2015)	United Kingdom	283	55.1	Youth	Range: 9–14	NA	7 days	Longitudinal	Weather
Harrison et al. (2017)	United Kingdom, Switzerland, Belgium, Australia, Denmark, Estonia, Norway, Madeira, United States	23,451	62.0	Youth	Range: 3–18	NA	7 days	Longitudinal	Weather
Hjorth et al. (2013)	Denmark	730	48.5	Youth	10.0 ± 0.6	NA	7 days	Longitudinal	Weather
Hoaas et al. (2019)	Norway, Denmark, Australia	168	42.9	Adults	Range: 60–73	NA	7 days	Longitudinal	Season
Hopkins et al. (2011)	United States	145	59.3	Youth	10.7 ± 0.3	NA	7 days	Longitudinal	Season
Hoppmann et al. (2017)	Canada	126	64.0	Older adults	71.9 ± 5	European: 62.0%; Asian: 36.0%; Other: 1.0%; Missing: 1.0%	10 days	Longitudinal	Weather
Hunter et al. (2019)	Canada	149	47.0	Adults	19 ± 1.9	White: 59.7%; Other: 40.3%	7 days	Longitudinal	Weather
Jehn et al. (2014)	Germany	15	40.0	Adults	66.7 ± 5.2	NA	6 months	Longitudinal	Season
Jones et al. (2017)	Canada	42	24.0	Older adults	77.4 ± 4.7	NA	7 days	Cross-sectional	Weather
Jones et al. (2017)	Canada	42	NA	Older adults	77.4 ± 4.7	NA	7 days	Longitudinal	Season, Weather
Katapally et al. (2016)	Canada	331	49.8	Youth	11.6 ± 1.1	NA	7 days	Longitudinal	Weather
Kharlova et al. (2020)	Norway	2015	50.6	Youth	9.5 ± 1.8	NA	6 days	Longitudinal	Season
Kimura et al. (2015)	Japan	39	56.0	Older adults	70.7 ± 3.2	NA	2 weeks	Longitudinal	Weather
King et al. (2011)	United Kingdom	480	49.1	Youth	NA	NA	7 days	Longitudinal	Season
Kolle et al. (2009)	Norway	1824	48.5	Youth	Range: 9–15	NA	4 days	Cross-sectional	Weather
Kong et al. (2020)	South Korea	555	43.8	Adults	61.1 ± 8.9	NA	7 days	cross-sectional	Weather
Koolhaas et al. (2017)	Netherlands	1200	52.3	Older adults	77.5 ± 5.0	NA	7 days	Cross-sectional	Season
Larouche et al. (2019)	Canada	1699	55.0	Youth	10.2 ± 1.0	NA	8 days	Cross-sectional	Season
Lewis et al. (2016)	Australia and Canada	953	57.0	Youth	10.6 ± 0.4	NA	7 days	Cross-sectional	Season
Ma et al. (2018)	Hong Kong	210	33.8	Adults	26.1 ± 8.7	NA	35 days	Longitudinal	Season
Martins et al. (2015)	Brazil	16	50.0	Adults	Range: 18–35	NA	6 days	Longitudinal	Weather
McCrorie et al. (2015)	Scotland	33	54.5	Youth	12.2 ± 0.3	NA	8 days	Cross-sectional	Weather
McKee et al. (2012)	Northern Ireland	85	38.8	Youth	Range: 4–5	NA	6 days	Longitudinal	Weather
McMurdo et al. (2012)	United Kingdom	547	54.0	Older adults	79 ± 8	NA	7 days	Cross-sectional	Weather
Mitchell et al. (2018)	United States	575	34.3	Adults	38.6 ± 0.1	Hispanic: 100.0%	1 day	Cross-sectional	Weather
Mitsui et al. (2010)	Japan	50	0.0	Adults	43.6 ± 10.8	NA	7 days	Longitudinal	Weather
Nagy et al. (2019)	United Kingdom	108	51.0	Youth	7.5 ± 0.5	Caucasian: 59.0%; South Asian: 41.0%	7 days	Cross-sectional	Season
Nakashima et al. (2019)	Japan	22	86.4	Older adults	75.1 ± 7.3	NA	7 days	Longitudinal	Season
Newman et al. (2009)	United States	500	100.0	Adults	57 ± 2.9	African-American: 13.2%	7 days	Cross-sectional	Season
Nilsen et al. (2019)	Norway	1154	49.0	Youth	4.7 ± 0.9	NA	14 days	Longitudinal	Season
O’Connell et al. (2013)	United Kingdom	46	72.0	Adults	41.7 ± 14.4	NA	7 days	Longitudinal	Season
Ogawa et al. (2018)	Japan	35	65.7	Adults	69.3 ± 5.3	NA	1 month	Longitudinal	Season
Oliver et al. (2011)	New Zealand	135	60.0	Youth	Range: 5–6	NA	8 days	Cross-sectional	Season
Pagels et al. (2016)	Sweden	179	48.6	Youth	11.1 ± 2.1	NA	5 days	Longitudinal	Season
Patnode et al. (2010)	United States	294	49.3	Youth	15.4 ± 1.7	Caucasian: 93.5%; Other: 6.5%	7 days	Cross-sectional	Season, weather
Pearce et al. (2012)	United Kingdom	482	52.0	Youth	Range: 8–10	NA	7 days	Longitudinal	Season
Pechova et al. (2019)	Czech Republic, Slovakia & Poland	83	89.7	Adults	65 ± 6	NA	8 days	Longitudinal	Season, Weather
Pelclova et al. (2010)	Czech Republic	13	84.6	Youth	15.6 ± 0.5	NA	10 months	Longitudinal	Weather
Prins and van Lenthe (2015)	Netherlands	43	52.5	Adults	NA	NA	7 days	Longitudinal	Weather
Rahman et al. (2019)	Canada	972	58.0	Youth	10.9 ± 0.4	NA	7 days	Cross-sectional	Season, weather
Remmers et al. (2017)	Australia	307	52.0	Youth	11.1 ± 0.7	NA	7 days	Longitudinal	Season, Weather
Ridgers et al. (2015)	Australia	326	50.3	Youth	10 ± 0.7	NA	7 days	Cross-sectional	Season
Ridgers et al. (2018)	Australia	326	50.3	Youth	Range: 8–11	NA	7 days	Longitudinal	Season
Robbins et al. (2013)	Canada	38	26.3	Adults	54 ± 7.0	NA	7 days	Cross-sectional	Weather
Rosenthal et al. (2020)	United States	266	58.4	Adults	52.1 ± 14	NA	NA	Cross-sectional	Weather
Rowlands et al. (2009)	United Kingdom	64	50.0	Youth	9.9 ± 0.3	NA	6 days	Longitudinal	Season
Sartini et al. (2017)	United Kingdom	1361	0.0	Older adults	78.5 ± 4.6	NA	7 days	Longitudinal	Weather
Schepps et al. (2018)	United States	16,741	100.0	Older adults	72.0 ± 5.7	NA	7 days	Cross-sectional	Weather
Sewell et al. (2010)	United Kingdom	95	41.0	Adults	65.5 ± 8.5	NA	2 days	Cross-sectional	Season
Shen et al. (2013)	United States	46	56.5	Youth	4.2 ± 0.2	Caucasian: 68.0%; Other: 32.0%	5 days	Cross-sectional	Weather
Silva et al. (2011)	Portugal	24	50.0	Youth	11.0 ± 1.5	NA	7 days	Longitudinal	Season
Sit et al. (2019)	Hong Kong	270	40.0	Youth	NA	NA	3 days	Longitudinal	Season
Stabell et al. (2020)	United States	38	10.5	Adults	Range: 50–75	Caucasian:81.6%; Other: 18.4%	24 weeks	Longitudinal	Weather
Sugino et al. (2012)	Japan	9	0.0	Adults	71.7 ± 8.3	NA	2 weeks	Cross-sectional	Season
Sumukadas et al. (2008)	Scotland	127	71.0	Older adults	78.6 ± 6.5	NA	7 days	Longitudinal	Season
Van Kann et al. (2016)	Netherlands	520	55.6	Youth	10.1 ± 0.7	NA	7 days	Longitudinal	Weather
Wang et al. (2017)	China	34	38.2	Adults	31 ± 10	NA	7 days	Longitudinal	Weather
Witham et al. (2014)	United Kingdom	547	54.3	Older adults	78.5 ± 7.7	NA	7 days	Longitudinal	Weather
Wong et al. (2020)	Mexico	559	49.0	Youth	4.8 ± 0.5	NA	7 days	Longitudinal	Weather
Wu et al. (2017)	United Kingdom	4051	55.7	Adults	67.4 ± 9.5	NA	7 days	Cross-sectional	Weather
Yildrim et al. (2012)	Belgium, Greece, Hungary, Netherlands, Switzerland	722	53.0	Youth	11.6 ± 0.9	NA	6 days	Cross-sectional	Weather
Zheng et al. (2019)	Hong Kong	740	40.9	Youth	14.7 ± 1.6	Chinese: 100.0%	7 days	Longitudinal	Weather

Approximately equal numbers of studies examined weather (77/144, 53.5%) and seasonality (67/144, 46.5%). Studies sampled youth (persons under age 18; 73/144, 50.7%), adults (44/144, 30.6%), and older adults (typically defined as over age 65; 27/144, 18.8%). Study designs were longitudinal (97/144, 67.4%) and cross-sectional (47/144, 32.6%). The modal duration of activity monitoring for movement behavior was 7 days (82/144, 56.9%) but ranged from 1 day to 2 years.

### Activity monitoring methods

Almost all studies measured physical activity (138/144, 95.2%) and fewer measured sedentary behavior (50/144, 34.7%). Table 2 summarizes characteristics of the devices used to measure physical activity and sedentary behavior. Physical activity was measured with research-grade accelerometers (121/144, 84.0%), pedometers (22/144, 15.3%), or global positioning system logger (1/144, 0.7%). Although most studies with accelerometer-based measurements used wearable devices, one study measured physical activity using the accelerometer contained within a smartphone [46].

**Table 2:** Characteristics of devices used to measure physical activity and sedentary behavior

Reference	Type of Device	Specific Device	Wear Location	Measure(s) of Activity
Aadland et al. (2018)	Accelerometer	ActiGraph GT3X+	NA	SB, LPA, MVPA, V
Aebi et al. (2020)	Accelerometer	ActiGraph wGT3X-BT	Hip	SB, LPA, MVPA
Aibar Solana et al. (2015)	Accelerometer	ActiGraph GT3X	NA	SB
Alande et al. (2019)	Pedometer	Kaden G-Sport Pocket Pedometer 793	Waist	V
Albrecht et al. (2020)	Accelerometer	ActiGraph wGT3X-BT	Wrist	V
Al-Mohannadi et al. (2016)	Pedometer	Omron HJ-720 ITC	NA	V
Arnardottir et al. (2017)	Accelerometer	ActiGraph GT3X+	Hip	SB, LPA, MVPA, V
Aspvik et al. (2018)	Accelerometer	ActiGraph GT3X+	Waist	V
Atkin et al. (2016)	Accelerometer	ActiGraph GT1M	Waist	SB, MVPA
Badland et al. (2011)	Pedometer	Yamax Digiwalker SW-200	Hip	V
Balish et al. (2017)	Pedometer	Yamax Digiwalker SW-200	Hip	V
Barkley and Herrmann (2017)	Accelerometer	ActiGraph GT3X	Waist	LPA, MVPA
Beighle et al. (2013)	Pedometer	MLS 205	Waist	V
Bejarano et al. (2019)	Accelerometer	ActiGraph wActi Sleep-BT	Wrist	SB, MVPA
Boutou et al. (2019)	Accelerometer	ActiGraph GT3X	Hip	MVPA, V
Brandon et al. (2009)	Accelerometer	ActiGraph GT1M	Torso	V
Bremer et al. (2019)	Pedometer	PiezoRX	Hip	V
Bringolf-Isler et al. (2009)	Accelerometer	ActiGraph AM7164	Waist	V
Brychta et al. (2016)	Accelerometer	ActiGraph GT3X	Hip	V
Buchowski et al. (2009)	Accelerometer	Tritrac-R3D	NA	SB, V
Button et al. (2020)	Accelerometer	Actical Z	Hip	LPA, MVPA
Carr et al. (2016)	Pedometer	Omron HJ-720 ITC	NA	V
Cepeda et al. (2018)	Accelerometer	GENEActiv	Wrist	SB, LPA, MVPA
Chang et al. (2020)	Accelerometer	ActiGraph GT3X	Hip	SB, LPA, MVPA, V
Clemes et al. (2011)	Pedometer	Yamax Digiwalker SW-200	Waist	V
Collings et al. (2020)	Accelerometer	ActiGraph GT3X+	Hip	SB, LPA, MVPA, V
Colom et al. (2019)	Accelerometer	GENEActiv	Wrist	MVPA
Cooper et al. (2010)	Accelerometer	ActiGraph GT1M	Waist	V
Cradock et al. (2009)	Accelerometer	TriTac-R3D	Hip	MVPA
Crowley et al. (2016)	Pedometer	Jawbone UP	Wrist	V
Cullen et al. (2017)	Accelerometer	ActiGraph GT3X+	NA	V
Davis et al. (2011)	Accelerometer	ActiGraph GT1M	Waist	LPA
de Vries et al. (2019)	Accelerometer	1. ActiWatch 2; 2. GENEActiv Original; 3. Sensewear Mini	1. Wrist; 2. Wrist; 3. Arm	SB, LPA, MVPA, V
Declòs-Alió et al. (2019)	Accelerometer	ActiGraph GT3X	NA	V
Deng and Fredriksen (2018)	Accelerometer	ActiGraph wGT3X-BT	Hip	MVPA
Dias et al. (2019)	Accelerometer	1. ActiGraph 7164; 2. ActiGraph GT1M; 3. ActiGraph 71256	Waist	SB, V, MVPA
Diaz et al. (2016)	Accelerometer	Actical	Waist	SB
Dill et al. (2014)	Accelerometer	ActiGraph GT3X	Hip	V, MVPA
Duncan et al. (2008)	Pedometer	New Lifestyles 2000	Waist	V
Edwards et al. (2015)	Accelerometer	RT3	Hip	SB, MVPA, V
Feinglass et al. (2011)	Accelerometer	ActiGraph GT1M	Hip	V
Goodman et al. (2012)	Accelerometer	RT3	Waist	MVPA
Goodman et al. (2014)	Accelerometer	1. ActiGraph 7164; 2. ActiGraph GT1M; 3. ActiGraph 71256	Waist	V
Gracia-Marco et al. (2013)	Accelerometer	ActiGraph GT1M	Back	SB, MVPA, V
Griew et al. (2010)	Accelerometer	ActiGraph GT1M	Hip	V
Hagströmer et al. (2014)	Accelerometer	ActiGraph 7164	Hip	SB, MVPA, V
Hamilton et al. (2009)	Pedometer	New Lifestyles Digi-Walker SW-200	NA	V
Harrison et al. (2011)	Accelerometer	ActiGraph GT1M	Hip	SB, V, MVPA
Harrison et al. (2015)	Accelerometer	ActiGraph GT1M	Hip	SB, MVPA, V
Harrison et al. (2017)	Accelerometer	1. ActiGraph 7164; 2. ActiGraph GT1M	NA	V
Hjorth et al. (2013)	Accelerometer	ActiGraph GT3X+	Hip	SB, MVPA, V
Hoaas et al. (2019)	Accelerometer	SenseWear Pro	Arm	SB, LPA, MVPA, V
Hopkins et al. (2011)	Accelerometer	ActiGraph GT1M	Hip	V
Hoppmann et al. (2017)	Accelerometer	ActiGraph GT3X	Hip	MVPA, V
Hunter et al. (2019)	Accelerometer	ActiGraph wGT3X-BT	Waist	SB, LPA, MVPA, V
Jehn et al. (2014)	Accelerometer	AiperMotion	Hip	V
Jones et al. (2017)	Accelerometer	ActiGraph GT1M	Waist	V
Jones et al. (2017)	Accelerometer	ActiGraph GT1M	Waist	V
Katapally et al. (2016)	Accelerometer	Actical	Waist	SB
Kharlova et al. (2020)	Accelerometer	ActiGraph wGT3X-BT	Hip	SB, MVPA
Kimura et al. (2015)	Pedometer	Kenz Lifecorder EX	Waist	V
King et al. (2011)	Accelerometer	1. ActiGraph 7164; 2. ActiGraph GT1M	Hip	SB, V
Kolle et al. (2009)	Accelerometer	ActiGraph MTI 7164	Waist	V
Kong et al. (2020)	Accelerometer	Fitbit Flex	Wrist	MVPA, V
Koolhaas et al. (2017)	Accelerometer	GENEActiv	Wrist	SB, LPA, MVPA
Larouche et al. (2019)	Pedometer	SC-Step Rx	NA	MVPA, V
Lewis et al. (2016)	Accelerometer	ActiGraph GT3X+	Hip	SB, MVPA
Ma et al. (2018)	Accelerometer	1. iPhones 5S ; 2. iPhone 6	NA	V
Martins et al. (2015)	Accelerometer	ActiGraph GT3X+	Hip	MVPA
McCrorie et al. (2015)	Accelerometer	activPAL	Thigh	V
McKee et al. (2012)	Pedometer	DigiWalker DW-2000	NA	V
McMurdo et al. (2012)	Accelerometer	RT3	Hip	V
Mitchell et al. (2018)	Accelerometer	Actical	NA	V
Mitsui et al. (2010)	Pedometer	Yamasa EM-180	NA	V
Nagy et al. (2019)	Accelerometer	ActiGraph GT3X+	Hip	SB, MVPA, V
Nakashima et al. (2019)	Accelerometer	LifeLyzer05 Coach	Back	LPA, MVPA, V
Newman et al. (2009)	Pedometer	Accusplit AE120	Hip	V
Nilsen et al. (2019)	Accelerometer	ActiGraph GT3X+	Hip	SB, LPA, MVPA, V
O’Connell et al. (2013)	Accelerometer	ActiGraph GT1M	Hip	SB, LPA, MVPA
Ogawa et al. (2018)	Accelerometer	Kenz Lifecorder GS	Waist	MVPA, V
Oliver et al. (2011)	Accelerometer	Mini-mitter	NA	MVPA
Pagels et al. (2016)	Accelerometer	ActiGraph GT3X+	NA	MVPA
Patnode et al. (2010)	Accelerometer	ActiGraph 7164	Hip	MVPA
Pearce et al. (2012)	Accelerometer	ActiGraph GT1M	Hip	V
Pechova et al. (2019)	Accelerometer	ActiGraph GT1M	Hip	SB
Pelclova et al. (2010)	Pedometer	Omron HJ-105	Hip	V
Prins and van Lenthe (2015)	GPS Logger	QStarz BT-Q1000XT	Hip	V
Rahman et al. (2019)	Pedometer	Omron HJ-720 ITC	Hip	V
Remmers et al. (2017)	Accelerometer	ActiGraph GT3X+	Hip	SB, LPA, MVPA
Ridgers et al. (2015)	Accelerometer	ActiGraph GT3X+	Hip	MVPA
Ridgers et al. (2018)	Accelerometer	ActiGraph GT3X+	Hip	LPA, MVPA
Robbins et al. (2013)	Accelerometer	ActiGraph GT1M	Hip	MVPA, V
Rosenthal et al. (2020)	Accelerometer	NA	NA	V
Rowlands et al. (2009)	Accelerometer	ActiGraph GT1M	Hip	MVPA, V
Sartini et al. (2017)	Accelerometer	ActiGraph GT3X	Hip	SB, LPA, MVPA, V
Schepps et al. (2018)	Accelerometer	ActiGraph GT3X+	Hip	SB, LPA, MVPA, V
Sewell et al. (2010)	Accelerometer	Gaehwiler Electronic Z80-32 k V1	Waist	V
Shen et al. (2013)	Accelerometer	ActiGraph GT1M	Hip	LPA, MVPA
Silva et al. (2011)	Accelerometer	ActiGraph MTI/CSA 7164	Hip	SB, MVPA
Sit et al. (2019)	Accelerometer	ActiGraph GT3X	Hip	SB, MVPA
Stabell et al. (2020)	Pedometer	Omron HJ-324 U	Waist	V
Sugino et al. (2012)	Accelerometer	1. Actimarker; 2. Dynaport Activity Monitor (DAM)	Waist	V
Sumukadas et al. (2008)	Accelerometer	RT3	Hip	V
Van Kann et al. (2016)	Accelerometer	Actiheart	Chest	LPA, MVPA
Wang et al. (2017)	Accelerometer	ActiGraph GT3X	Waist	MVPA, V
Witham et al. (2014)	Accelerometer	RT3	Waist	V
Wong et al. (2020)	Accelerometer	ActiGraph GT3X+	Hip	SB
Wu et al. (2017)	Accelerometer	ActiGraph GT1M	Wrist	SB, V
Yildrim et al. (2012)	Accelerometer	1. ActiGraph ActiTrainer; 2. ActiGraph GT1M; 3. ActiGraph GT3X	Waist	SB, LPA, MVPA, V
Zheng et al. (2019)	Accelerometer	activPAL	Thigh	SB, MVPA

*Note. V* Physical activity volume, *LPA* light-intensity physical activity duration, *MVPA* moderate-to-vigorous intensity physical activity duration, *SB* sedentary behavior duration

**Fig. 3:**
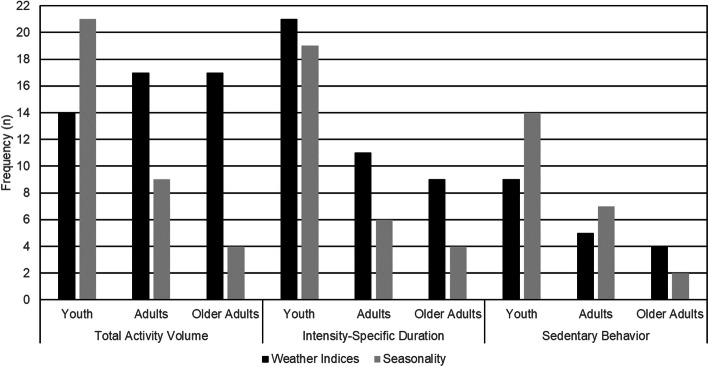
Movement behavior measures in studies examining seasonality and weather indices across the lifespan

Physical activity measures included volume (i.e., step counts, total accelerometer counts) and intensity-specific durations. Volume measures represent the total amount of energy expended in physical activity whereas intensity-specific durations represent how individuals allocate their time to more and less effortful forms of physical activity. Figure 3 summarizes the frequency of studies using volume and intensity-specific duration measures in samples of youth, adults, and older adults. Seasonal differences in physical activity were more frequently estimated in youth than adults or older adults. Weather indices associated with physical activity were studied most frequently in youth, and approximately equally in adults and older adults.

Sedentary behavior was typically classified using accelerometer cut-points that included < 50, < 100, ≤100, < 150, < 175, and < 200 counts per minute [33, 38, 39, 47–70]. Other sedentary behavior classifications were based on accumulating less than 122 counts per minute, less than or equal to 328 counts per minute [71], less than 800 vector magnitude counts per minute [72], less than 820 vector magnitude counts per minute [73, 74], less than 1110 vector magnitude counts per minute [75, 76], falling below activity registering at 1.0 [77] or 1.5 [78–80] metabolic equivalents to task (METs). Four studies in three papers did not report how sedentary behavior was defined [30, 34, 81].

### Seasonal differences in physical activity and movement-related behaviors

Table 3 summarizes seasonal differences in a variety of movement-related behaviors. Studies either compared two seasons (29/67, 43.3%), three seasons (9/67, 13.4%), or four seasons (25/67, 37.3%). Four studies did not report their definition of season (4/67, 6.0%) [86, 87, 103]. Winter (64/67, 95.5%) and summer (51/67, 76.1%) were studied most frequently, with spring (40/67, 59.7%) and autumn (36/67, 53.7%) receiving less attention. Studies comparing physical activity and sedentary behavior between seasons sampled youth (40/67, 59.7%), adults (17/67, 25.4%) and older adults (10/67, 14.9%). Studies of seasonal differences measured volume (47/67, 70.1%), moderate-to-vigorous intensity physical activity (MVPA) duration (37/67, 55.2%), light-intensity physical activity (LPA) duration (19/67, 28.4%), and sedentary behavior (25/67, 37.3%).

**Table 3:** Seasonal differences in movement-related behaviors

Season	Reference Season	Physical Activity Volume	Light-Intensity Physical Activity	Moderate-to-Vigorous Intensity Physical Activity	Sedentary Behavior
Winter	> Summer	1 [82]	0	1 [79]	10 [30, 47, 51, 52, 58, 67, 72, 74, 75, 79]
	= Summer	6 [65, 72, 74, 80, 83, 84]	6 [52, 55, 79, 80, 85]	7 [65, 67, 74, 80, 85]	3 [65, 74, 80]
	< Summer	23 [28, 31, 44, 52, 74–77, 86–99]	6 [53, 67, 74, 89, 100]	11 [31, 51–53, 58, 72, 89, 100–102]	0
	Evidence Grade	Strong	Moderate	Strong	Strong
	> Spring	0	0	0	11 [51, 52, 54, 55, 67–69, 72, 74, 79]
	= Spring	7 [29, 49, 65, 68, 80, 84, 98]	7 [30, 52, 74, 80, 100]	10 [49, 53, 55, 65, 67, 68, 79, 80]	5 [53, 65, 68, 77, 80]
	< Spring	15 [29, 52, 54, 68, 72, 74, 89, 90, 103–107]	7 [53–55, 64, 67, 79, 89]	12 [33, 52, 54, 68, 72, 74, 89, 100, 101, 105]	0
	Evidence Grade	Strong	Moderate	Strong	Strong
	> Autumn	0	2 [79, 100]	1 [79]	3 [52, 67, 74]
	= Autumn	7 [65, 68, 72, 74, 80, 84, 98]	4 [52, 55, 74, 80]	12 [49, 52, 65–68, 72, 74, 80, 101]	8 [65, 66, 68, 72, 74, 79, 80]
	< Autumn	5 [40, 52, 66, 74, 104]	5 [32, 66, 67, 74]	4 [32, 40, 100]	0
	Evidence Grade	Moderate	Limited	Strong	Moderate
Spring	> Autumn	5 [29, 52, 65, 90]	1 [67]	5 [33, 52, 65, 79]	0
	= Autumn	6 [29, 49, 80, 84, 98, 105]	5 [30, 52, 64, 79, 80]	7 [30, 49, 55, 67, 80, 105]	5 [53, 55, 65, 79, 80]
	< Autumn	1 [104]	1 [30]	0	4 [52, 63, 67, 77]
	Evidence Grade	Moderate	Limited	Moderate	Limited
	> Summer	2 [52, 82]	1 [79]	2 [33, 79]	0
	= Summer	6 [65, 80, 84, 98, 105, 108]	7 [30, 52, 64, 67, 80, 108]	10 [30, 49, 52, 55, 65, 67, 80, 105, 108]	8 [53, 55, 65, 67, 69, 79, 80, 108]
	< Summer	2 [87, 104]	0	1 [41]	2 [52, 77]
	Evidence Grade	Limited	Moderate	Strong	Moderate
Summer	> Autumn	1 [87]	1 [67]	0	0
	= Autumn	5 [52, 65, 80, 84, 98]	2 [52, 79]	5 [52, 65, 67, 79, 80]	4 [52, 65, 69, 80]
	< Autumn	1 [82]	0	0	2 [67, 79]
	Evidence Grade	Limited	Not Assignable	Moderate	Limited

#### Physical activity volume

Studies comparing volume between seasons sampled youth (29/47, 61.7%), adults (13/47, 27.7%) and older adults (5/47, 10.6%). The most common seasonal comparisons of volume involved winter vs. summer (30/47, 63.8%), winter vs. spring (22/47, 46.8%), spring vs. autumn (12/47, 25.5%), and winter vs. autumn (12/47, 25.5%).

Strong evidence indicated that physical activity volume was greater in summer than winter (23/30, 76.7%), although one study found it was greater in winter than summer (1/30, 3.3%). Six studies (20.0%) comparing volume in winter and summer yielded null results. Strong evidence also indicated that physical activity volume was consistently greater in spring than winter (15/22, 68.2%), but seven studies indicated no difference (7/22, 31.8%). No studies found evidence of greater volume in winter than spring.

Moderate evidence indicated mixed findings for physical activity volume between spring and autumn with most studies revealing either greater volume in spring than autumn (5/12, 41.7%) or no differences in volume (6/12, 50.0%). One study reported greater volume in autumn than spring (1/12, 8.3%). Moderate evidence indicated mixed findings for physical activity volume between autumn and winter with some studies indicating no difference (7/12, 58.3%) and others indicating great volume in autumn than winter (5/12, 41.7%). No studies found evidence of greater physical activity volume in winter than autumn.

Limited evidence compared physical activity volume in spring and summer were equivocal: six studies indicated no difference in volume between summer and spring (6/10, 60%) but the remaining studies were evenly split between greater volume in summer (2/10, 20.0%) and spring (2/10, 20.0%). Limited evidence compared physical activity volume between autumn and summer. Findings generally indicated no difference (5/7, 71.4%) with the exception of one study indicating greater volume in autumn than summer (1/7, 14.3%) and another study indicating greater volume in summer than autumn (5/7, 14.3%).

#### Intensity-specific physical activity duration

Seasonal comparisons more frequently involved the duration of MVPA (37/37, 100.0%) than LPA (20/37, 54.1%). Studies comparing MVPA duration between seasons sampled youth (25/37, 67.6%), adults (6/37, 16.2%), and older adults (6/67 16.7%). Seasonal comparisons of LPA duration sampled youth (11/20, 55.0%), older adults (6/20, 30.0%), and adults (3/20, 15.0%).

As shown in Table 3, the most common seasonal comparisons of intensity-specific behavior durations involved winter vs. spring (overall: 22/37 [59.5%]; MVPA: 22/22 [100%]; LPA: 14/22 [63.6%]; winter vs. summer (overall: 19/37 [51.4%]; MVPA: 19/19 [%]; LPA: 12/19 [63.2%]), winter vs. autumn (overall: 17/37 [45.9%]; MVPA: 17/17 [100%]; LPA: 11/17 [64.7%]), spring vs. summer (overall: 13/37 [35.1%]; MVPA: 13/13 [100%]; LPA: 8/13 [61.5%]), and spring vs. autumn (overall: 12/37 [32.4%]; MVPA: 12/12 [100%]; LPA: 7/12 [58.3%]).

Strong evidence indicated either greater MVPA in spring than winter (12/22, 54.5%) or no difference between these seasons (10/22, 44.5%). No studies found greater MVPA duration in winter than spring. Based on moderate evidence, findings about LPA were mixed: half of the studies reported greater LPA in spring than winter (7/14, 50.0%) and half reported no difference in LPA between spring and winter (7/14, 50.0%). Strong evidence indicated that MVPA duration was generally greater in summer than winter (11/19, 57.9%). Six studies found no difference between these seasons (6/19, 20%) and one study found winter activity greater than summer (1/30, 3.3%). Based on moderate evidence, studies indicated either greater LPA in summer than winter (6/12, 50.0%) or no difference (6/12, 50.0%). Strong evidence found mixed findings for seasonal differences in MVPA between autumn and winter, either indicating no difference (12/17, 70.6%) or greater MVPA in autumn than winter (4/17, 23.5%). One study demonstrated greater MVPA duration in autumn compared to winter (1/17, 5.9%). Based on limited evidence, LPA was either greater in autumn than winter (5/11, 45.5%), not different between these seasons (4/11, 36.6%), or greater in winter than autumn (2/11, 18.2%). Strong evidence indicated that MVPA duration typically did not differ between summer and spring (10/13, 76.9%), but two studies revealed greater MVPA durations in summer (2/13, 15.3%) and another revealed greater MVPA duration in spring (1/13, 7.7%). Moderate evidence indicated that LPA generally did not differ between summer than spring (7/8, 87.5%) but one study indicated greater LPA in spring than summer (1/8, 12.5%).

Moderate evidence indicated mixed findings from comparisons of MVPA between spring and autumn, either indicating no differences (7/12, 58.3%) or greater MVPA in spring than autumn (5/12, 41.7%). No study demonstrated greater MVPA duration in autumn compared to spring. Based on limited evidence, LPA generally did not differ between spring and autumn (5/7, 71.4%); however, one study indicated greater LPA in spring than autumn (1/7, 14.3%) and another study indicated greater LPA in autumn than spring (1/7, 14.3%). Moderate evidence indicated that MVPA duration did not differ between summer and autumn in five studies (5/5, 100%). A grade was not assignable for LPA comparisons between summer and autumn.

#### Sedentary behavior

Studies comparing sedentary behavior between seasons sampled youth (14/23, 60.9%), adults (7/23, 30.4%) and older adults (2/23, 8.7%). The most common seasonal comparisons of volume involved winter vs. spring (16/23, 69.6%), winter vs. summer (13/23, 56.5%), winter vs. autumn (11/23, 47.8%), spring vs. summer (10/23, 43.5%), spring vs. autumn (9/23, 39.1%), and summer vs. autumn (6/23, 26.1%).

Strong evidence indicated that sedentary behavior was generally greater in winter than spring (11/16, 68.8%) although some studies indicated no difference (5/16, 31.3%). Strong evidence indicated that sedentary behavior was generally greater in winter than summer (10/13, 76.9%) with a few studies indicating no difference (3/13, 23.1%). Moderate evidence indicated that sedentary behavior did not vary between autumn and winter (8/11, 72.7%) but a few studies indicated greater sedentary behavior in winter than autumn (3/11, 27.3%). Moderate evidence indicated that sedentary behavior generally did not differ between spring and summer (8/10, 80%) but two studied indicated greater sedentary behavior during summer (2/10, 20%). Limited evidence also indicated that sedentary behavior either did not differ between spring and autumn (5/9, 55.6%) or was greater in autumn than spring (4/9, 44.4%). Limited evidence indicated that sedentary behavior either did not differ between summer and autumn (4/6, 66.7%) or was greater in autumn than summer (2/6, 33.3%).

### Specific weather indices associated with movement-related behaviors

Weather indices were typically collected from regional weather stations or national institutes (73/77, 94.8%); few studies used self-reported weather indices (3/77, 3.9%) or did not specify where weather indices were collected (1/77, 1.3%). Associations between specific indices and behavior examined in five or more studies are discussed below; all available results are summarized in Table 4. The weather indices are defined in Appendix 2. The most frequently-reported weather indices included temperature (60/77, 77.9%), precipitation (58/77, 75.3%), photoperiod (33/77, 42.9%), wind speed (20/77, 26.0%), humidity (16/77, 20.8%), snow (12/77, 15.6%), and cloud coverage (7/77, 9.1%). No grade was assigned to research on barometric pressure, visibility, wind chill, or air quality due to insufficient evidence.

**Table 4:** Associations between movement-related behaviors and specific weather indices

Weather Index	Finding	Physical Activity Volume	Light-Intensity Physical Activity	Moderate-to-Vigorous Intensity Physical Activity	Sedentary Behavior
Temperature	Positive Associations	19 [35–38, 40, 43, 47, 70, 88, 107–114]	4 [47, 64, 70, 115]	11 [35, 39, 42, 47, 57, 73, 79, 81, 110, 116]	2 [38, 81]
	Null Associations	13 [28, 35–37, 61, 117–122]	4 [34, 56, 116]	14 [34, 35, 40, 42, 56, 61, 70, 120, 121, 123, 124]	7 [34, 63, 70, 73, 81, 115]
	Negative Associations	6 [38, 46, 125–128]	0	3 [81, 122, 124]	9 [38, 39, 47, 60–63, 71, 109]
	Evidence Grade	Strong	Limited	Strong	Moderate
Precipitation	Positive Associations	1 [28]	0	2 [79, 123]	12 [38, 39, 48, 60, 61, 63, 71, 73, 81, 109, 129]
	Null Associations	5 [37, 70, 114, 118]	3 [34, 56, 70]	15 [35, 39, 42, 56, 70, 81, 121, 122, 124, 130, 131]	7 [34, 39, 62, 63, 70, 81]
	Negative Associations	25 [28, 36–38, 40, 43, 46, 48, 57, 61, 88, 109, 111–113, 119, 121, 122, 125, 127, 131, 132]	3 [34, 57, 116]	15 [34, 40, 48, 57, 61, 73, 81, 109, 116, 124, 129, 133, 134]	0
	Evidence Grade	Strong	Limited	Strong	Strong
Wind Speed	Positive Associations	0	0	1 [39]	2 [48, 60]
	Null Associations	6 [28, 36, 40, 43]	2 [34]	3 [34, 40]	4 [34, 39]
	Negative Associations	6 [36, 46, 48, 109, 113, 127]	0	3 [39, 48, 135]	0
	Evidence Grade	Moderate	Not Assignable	Limited	Limited
Photoperiod	Positive Associations	15 [38, 46–48, 50, 72, 78, 88, 109, 114, 125, 127, 134, 136, 137]	4 [34, 47, 64, 78]	9 [34, 48, 50, 72, 73, 78, 133, 135]	1 [63]
	Null Associations	6 [43, 113, 118, 121, 122]	1 [34]	6 [47, 79, 81, 121, 122]	3 [34, 81]
	Negative Associations	0	0	0	10 [38, 47, 48, 50, 63, 71–73, 78, 81]
	Evidence Grade	Strong	Limited	Strong	Strong
Snow	Positive Associations	0	0	0	0
	Null Associations	3 [111, 114, 125]	1 [116]	4 [39, 42, 116]	0
	Negative Associations	4 [38, 88, 93]	1 [64]	1 [40]	0
	Evidence Grade	Limited	Not Assignable	Limited	Not Assignable
Cloud Coverage	Positive Associations	1 [127]	0	0	0
	Null Associations	2 [36, 37]	0	0	0
	Negative Associations	2 [36, 37]	0	2 [41, 135]	0
	Evidence Grade	Limited	Not Assignable	Not Assignable	Not Assignable
Humidity	Positive Associations	0	0	0	2 [78, 81]
	Null Associations	0	1 [34]	6 [39, 81, 124]	5 [34, 39, 81]
	Negative Associations	5 [78, 107, 126, 127]	2 [34, 78]	6 [34, 78, 79, 81, 124]	0
	Evidence Grade	Moderate	Not Assignable	Moderate	Limited
Visibility	Positive Associations	1 [73]	0	0	0
	Null Associations	0	1 [34]	0	2 [34]
	Negative Associations	1 [127]	1 [34]	1 [34]	0
	Evidence Grade	Not Assignable	Not Assignable	Not Assignable	Not Assignable
Barometric Pressure	Positive Associations	1 [127]	0	0	0
	Null Associations	0	0	0	0
	Negative Associations	0	0	0	0
	Evidence Grade	Not Assignable	Not Assignable	Not Assignable	Not Assignable
Wind Chill	Positive Associations	1 [73]	0	0	0
	Null Associations	0	0	0	0
	Negative Associations	0	0	0	0
	Evidence Grade	Not Assignable	Not Assignable	Not Assignable	Not Assignable
Air Quality	Positive Associations	0	0	0	0
	Null Associations	1 [120]	0	1 [120]	0
	Negative Associations	1 [138]	0	0	0
	Evidence Grade	Not Assignable	Not Assignable	Not Assignable	Not Assignable

#### Temperature

Associations between temperature and movement-related behaviors were estimated in youth (24/60, 40.0%), adults (23/60, 38.3%), and older adults (13/60, 21.7%). The most common behavioral measure in these studies was physical activity volume (38/60, 61.7%), followed by intensity-specific physical activity duration (overall: 30/60 [50.0%]; MVPA: 28/30 [93.3%]; LPA: 8/30 [26.7%]) and sedentary behavior (18/60, 30.0%).

Associations between temperature and physical activity volume were more often positive (19/38, 50.0%) than null (13/38, 34.2%) or negative (6/38, 15.8%). No studies explicitly tested for curvilinear relations, but four studies noted an inverted-U pattern in which volume was lower during normatively warmer or colder days [38, 78, 117, 125]. Additional negative associations with volume were found when extreme temperatures were present [126, 127]. This evidence was graded as strong in favor of an inverted-U relation between temperature and physical activity volume.

Moderate evidence indicated that associations between temperature and MVPA duration were more often null (14/28, 50.0%) or positive (11/28, 39.3%) than negative (3/28, 10.7%). Limited evidence suggested that LPA exhibited a similar pattern with studies showing either a null (4/8, 50.0%) or positive (4/8, 50.0%) association with temperature. Moderate evidence indicated that sedentary behavior exhibited more negative associations with temperature (9/18, 50.0%) than null (7/18, 38.9%) or positive associations (2/18, 11.1%).

#### Precipitation

Associations between precipitation and movement-related behaviors were estimated in youth (28/58, 48.3%), adults (21/58, 36.2%) and older adults (9/58, 15.5%). Precipitation was most frequently studied in relation with physical activity volume (31/58, 53.4%) followed by intensity-specific physical activity durations (overall: 32/58 [55.2%]; MVPA: 32/32 [100%]; LPA: 6/32 [18.8%]) and sedentary behavior (19/32, 32.8%).

Strong evidence indicated that associations between precipitation and physical activity volume were largely negative (25/31, 80.6%) with a few null (5/31, 16.1%) and one positive (1/31, 3.2%) association. Strong evidence indicated that the association between precipitation and MVPA durations were mixed with negative (15/32, 46.9%) and null (15/32, 46.9%) results. Two studies reported a positive association between precipitation and MVPA duration (2/32, 6.3%). Limited evidence indicated that precipitation and LPA had either a negative (3/6, 50.0%) or null (3/6, 50.0%) association. Moderate evidence indicated a mix of positive (12/19, 63.2%) and null associations (7/19, 36.8%) between precipitation and sedentary behavior. No studies reported a negative association between precipitation and sedentary behavior.

#### Photoperiod

Associations between the photoperiod and movement-related behaviors were estimated in youth (14/33, 42.4%), adults (10/33, 30.3%) and older adults (9/33, 27.3%). These studies reported associations between the photoperiod and measures of physical activity volume (21/33, 63.6%), intensity-specific physical activity duration (overall: 19/33 [57.6%]; MVPA: 15/19 [78.9%], LPA: 5/19 [26.3%]) and sedentary behavior (14/33, 42.4%).

Strong evidence indicated that associations between photoperiod and physical activity volume were mostly positive (15/21, 71.4%) with a few null associations (6/21, 28.6%). No studies reported a negative association between photoperiod and physical activity volume. Strong evidence indicated that associations between the photoperiod and MVPA duration were mostly positive (9/15, 60.0%) or null (6/15, 40.0%). No studies reported a negative association between photoperiod and MVPA duration. Limited evidence suggested a positive association between photoperiod and LPA duration (4/5, 80.0%) with a single study indicating a null association (1/5, 20.0%). Strong evidence indicated that the photoperiod and sedentary behavior had a negative association (10/14, 71.4%), with a few studies indicating either null (3/14, 21.4%) or positive associations (1/14, 7.1%).

#### Wind speed

Associations between wind speed and movement-related behaviors were estimated in youth (8/20, 40.0%), older adults (7/20, 35.0%), and adults (5/20, 25.0%). These studies reported associations between wind speed and total physical activity volume (12/20, 60.0%) intensity-specific physical activity durations (overall: 7/20 [35.0%]; MVPA: 7/7 [100%]; LPA: 3/7 [42.9%]), and sedentary behavior (6/20, 35.0%).

Moderate evidence indicated that associations between wind speed and physical activity volume were a mix of null (6/12, 50.0%), and negative associations (6/12, 50.0%). No study reported a positive association between wind speed and volume.

Limited evidence indicated that wind speed and MVPA durations demonstrated mostly null (3/7, 42.9%) or negative associations (3/7, 42.9%]), although one study found a positive association (1/7, 14.3%). A grade was not assignable for evidence on wind speed and LPA. Limited evidence indicated that wind speed and sedentary behavior primarily displayed null (4/6, 66.7%) or positive associations (2/6, 33.3%); no studies reported a negative association.

#### Humidity

Associations between humidity and movement-related behaviors were estimated in adults (7/16, 43.8%), youth (5/16, 31.3%), and older adults (4/16, 25.0%). Studies including humidity frequently examined measures of physical activity volume (5/16, 31.3%), followed in frequency by intensity-specific physical activity duration (overall: 12/16 [75.0%]; MVPA: 12/12 [100%]; LPA: 3/12 [25.0%], sedentary behavior: 7/16 [43.8%]).

Moderate evidence indicated a consistently negative association between humidity and physical activity volume (5/5, 100.0%). Moderate evidence indicated a mix of negative (6/12, 50.0%) and null (6/12, 50.0%) associations between humidity and MVPA. A grade was not assignable to evidence linking humidity and LPA. Limited evidence suggested a null association between humidity and sedentary behavior (5/7, 71.4%) although some studies found a positive association (2/7, 28.6%).

#### Snow

Associations between snow and movement-related behaviors were estimated in youth (5/12, 41.7%), adults (3/12, 25.0%), and older adults (4/12, 33.3%). Studies including snow frequently examined measures of physical activity volume (7/12, 58.3%), followed in frequency by intensity-specific physical activity duration (overall: 6/12 [50.0%]; MVPA: 5/6 [83.3%], LPA: 2/6 [33.3%]).

Limited evidence on snow and physical activity volume was split between negative (4/7, 57.1%) and null (3/7, 42.9%) associations. No study examining snow and volume demonstrated a positive association. Limited evidence on snow and MVPA duration was split between null (4/5, 80.0%) and negative (1/5, 20.0%) associations. A grade was not assignable to evidence linking snow with either LPA or sedentary behavior.

#### Cloud coverage

Associations between cloud coverage and movement-related behaviors were estimated in youth (4/7, 57.1%), adults (1/7, 14.3%), and older adults (2/7, 28.6%). Studies including cloud coverage frequently examined measures of physical activity volume (5/7, 71.4%), followed in frequency by intensity-specific physical activity duration (overall: 2/7 [28.6%]; MVPA: 2/2 [100%], LPA: 0/2 [0.0%]).

Limited evidence indicated that cloud coverage and physical activity volume exhibit a negative (2/5, 40.0%) or null associations (2/5, 40.0%). One study found a positive association between cloud coverage and physical activity volume (1/5, 40.0%). A grade was not assignable to evidence linking cloud coverage and durations of MVPA, LPA, or sedentary behavior.

## Discussion

The present review summarized 144 studies from 110 articles with 118,189 participants from 30 countries. It updates conclusions from two seminal reviews based on a total of over 60 studies on over 300,000 participants from approximately 18 countries [10, 11]. In those reviews, studies on seasonal differences in physical activity greatly outnumbered studies on weather correlates. Over the past decade, attention has been divided more equally between seasonal differences in and weather correlates of movement behaviors. This review also extended the prior reviews by capturing the spectrum of movement behaviors ranging from physical activity volume to intensity-specific durations to sedentary behavior. Collectively, these reviews establish how key features of the natural environment are linked with a variety of movement behaviors.

Physical activity volume and MVPA duration were the most frequent measures of movement behavior. Consistent with prior work, winter and summer were marked by the lowest and greatest movement behavior, respectively [10, 11]. The present review extended those conclusions by documenting a trend in favor of greater physical activity volume and MVPA in spring than autumn. Comparisons of spring vs summer and summer vs autumn largely revealed no differences. Overall, the pattern of seasonal differences in physical activity volume and MVPA duration resembled a sinusoidal pattern and corresponded with both fluctuations in temperature and the waxing and waning photoperiod across the calendar year.

One contribution of the present review was that it examined a broad spectrum of movement behaviors that contribute to physical activity volume. MVPA duration has enjoyed a privileged status in the scientific literature because it has the strongest connections with health benefits [24]. MVPA also exhibited the clearest pattern of relations with seasonality and specific weather indices, including temperature, precipitation, and photoperiod. Thus, MVPA may provide one pathway by which the natural environment gets “under the skin” to affect health [139, 140].

Moving the needle on population-level MVPA has proven to be difficult [141, 142]. Far more time is spent in LPA than MVPA and LPA is a greater contributor to physical activity volume for most people [143]. Recent work has established unique health benefits from LPA after adjusting for MVPA [144]. As a consequence, LPA is a desirable substitute for prolonged sedentary behavior when MVPA is not feasible. Although a trend for increased LPA in summer and spring compared to winter was observed in the literature, LPA in the spring did not differ from LPA in the summer or autumn. In the interest of understanding how the natural environment facilitates or inhibits movement, this common form of physical activity should be a priority measure in future research examining seasonal differences and weather correlates.

Sedentary behavior was consistently greater in winter than spring or summer. Weather has been cited as a barrier to physical activity that facilitates sedentary behavior [8, 9]. This study extended previous work by reviewing how specific weather indices, as opposed to perceptions of the weather, are associated with sedentary behavior. People engage in more sedentary behavior on shorter days (photoperiod), when precipitation is greater, and when temperatures are lower. These findings revealed that weather is likely to be a third variable influencing the entire spectrum of movement-related behaviors.

These findings have two major implications. First, although the studies reviewed here were necessarily observational, they can inform behavioral interventions. Weather conditions that may serve as actual, as well as perceived, barriers to physical activity (e.g., too hot, cold, rainy or snowy, windy, or shorter days). Current or forecasted weather conditions may be useful for providing contextual information about opportunities for activity that could inform just-in-time interventions for movement behaviors. For example, users could be prompted to develop coping plans for exercise in adverse weather conditions and then reminded of those plans when adverse weather conditions were expected. Digital tools could also extend work on person-specific physical activity interventions by learning how to identify user-specific preferred weather patterns for movement behaviors and prompt users to ensure they capitalize on their preferred conditions to be active [145].

At a more general level, the impact of seasonal differences in movement behaviors on the implementation and evaluation of physical activity promotion programs should be considered when interpreting ambulatory behavior changes. Lifestyle physical activity intervention evaluations often last 1–6 months, and few last 12 months, so baseline and follow-up assessments are often conducted during different seasons. Seasonal influences on activity levels are typically allocated to the error term of statistical models but may be informative to include as a covariate or moderator of intervention effects. For example, including season as a moderator could reveal if interventions work better when days are lengthening (winter to summer) or when days are shortening (summer to winter).

Second, climate change is increasing the frequency of extreme weather conditions. Climatic zones that are currently favorable for movement behaviors may become inhospitable, inhibit physical activity, and contribute to health disparities. Some have speculated that climate change will increase migration as people seek to preserve an adaptive environmental niche [146]. Population-level data on movement behaviors could be investigated as a leading indicator of future health or migration due to climate change.

This review had limitations as well. The search was conducted using three databases and limited to English language publications. It is possible that studies were missed if they were published in journals not indexed by PubMed, CINAHL, or SPORTDiscus, or were written in languages other than English. All data were observational so strong causal inferences are not possible. Results were obtained from 30 countries on five continents but western and high-income countries were overrepresented in the data. Low- and middle-income countries are home to over 80% of the world’s population and four times as many deaths are attributed to physical inactivity in those countries than in high-income countries [147, 148]. Conclusions may not generalize equally well to all regions, climatic zones, or economic strata. Most studies did not report on race or ethnicity so it is unclear how physical, social or cultural differences influence seasonal differences or relations between weather and movement behaviors. Devices were used to obtain measures of physical activity volume and intensity-specific duration – primarily MVPA - but some activity types do not lend themselves to accurate measurement with devices (e.g., cycling, swimming) or are sub-optimal with devices attached at the waist (e.g., sedentary behavior). Devices also provide no insights into the specific domains of physical activity (e.g., occupational, transport-related, occupational, domestic) or sedentary behavior (e.g., reading, screen time, socializing, eating). The available literature has focused almost exclusively on aggregated weather summaries during monitoring periods. Yet weather is dynamic so those summaries may not generalize to within-person change processes [149]. Additionally, physiological or psychological differences in environmental tolerances likely exist. Some people, for example, will be more heat tolerant or may simply enjoy running in the rain. Person-specific models of physical activity under different weather conditions could shed light on these dynamics [145]. Finally, the review focused on 11 common weather indices but other indices may also be relevant.

## Conclusions

In sum, this review established consistent patterns of seasonal and weather-specific differences in physical activity and sedentary behavior. People tend to be most active in summer and least active in winter. This pattern coincides with temperature and photoperiod cycles. Weather may also influence comfort with physical activity as indicated by associations with precipitation and wind speed. These findings can inform physical activity promotion initiatives in the short-term and may have long-term implications for environmental influences on human health during the climate crisis.

## Supplementary Information

**Additional file 1.**

## Data Availability

All data generated or analyzed during this study are included in this published article and its supplementary information files.

## Appendix A

**Table 5:** Search Methods | PubMed

**Search terms:** ((“Exercise”[MeSH] OR “Exercises”[tiab] OR “Exercising”[MeSH] OR “Exercising”[tiab] OR “Physical activities”[MeSH] OR “Physical activities”[tiab] OR “Physical activity”[MeSH] OR “Physical activity”[tiab] OR “Physical fitness”[MeSH] OR “Physical fitness”[tiab] OR “Recreation”[MeSH] OR “Recreation”[tiab] OR “Run”[MeSH] OR “Run”[tiab] OR “Running”[MeSH] OR “Running”[tiab] OR “Walk”[MeSH] OR “Walk”[tiab] OR “Walking”[MeSH] OR “Walking”[tiab]) OR (“Computer game”[MesH] OR “Computer game”[tiab] OR “Computer games”[MeSH] OR “Computer games”[tiab] OR “Computer usage”[MeSH] OR “Computer usage”[tiab] OR “Computer use”[MeSH] OR “Computer use”[tiab] OR “Physically inactive”[MeSH] OR “Physically inactive”[tiab] OR “Screen time”[MeSH] OR “Screen time”[tiab] OR “Sedentarism”[MeSH] OR “Sedentarism”[tiab] OR “Sedentary behavior”[MeSH] OR “Sedentary behavior”[tiab] OR “TV viewing”[MeSH] OR “TV viewing”[tiab] OR “TV watching”[MeSH] OR “TV watching”[tiab] OR “Video game”[MeSH] OR “Video game”[tiab] OR “Video games”[MeSH] OR “Video games”[tiab] OR “Video gaming”[MeSH] OR “Video gaming”[tiab])) AND (“Accelerometer”[MeSH] OR “Accelerometer”[tiab] OR “Accelerometry”[MeSH] OR “Accelerometry”[tiab] OR “Pedometer”[MeSH] OR “Pedometer”[tiab] OR “Steps”[MeSH] OR “Steps”[tiab]) AND ((“Season”[MeSH] OR “Season”[tiab] OR “Seasonal”[MeSH] OR “Seasonal”[tiab] OR “Seasons”[MeSH] OR “Seasons”[tiab]) OR (“Atmospheric Pressure”[MesH] OR “Atmospheric Pressure”[tiab] OR “Cloud”[MeSH] OR “Cloud”[tiab] OR “Cloud Cover”[MeSH] OR “Cloud Cover”[tiab] OR “Clouds”[MeSH] OR “Clouds”[tiab] OR “Cloudy”[MeSH] OR “Cloudy”[tiab] OR “Fog”[MeSH] OR “Fog”[tiab] OR “Heat index”[MeSH] OR “Fog”[tiab] OR “Humid”[MeSH] OR “Humid”[tiab] OR “Humidity”[MeSH] OR “Humidity”[tiab] OR “Ice”[MeSH] OR “Ice”[tiab] OR “Lightning”[MeSH] OR “Lightning”[tiab] OR “Overcast”[MeSH] OR “Overcast”[tiab] OR “Precipitation”[MeSH] OR “Precipitation”[tiab] OR “Rain”[MeSH] OR “Rain”[tiab] OR “Saturation”[MeSH] OR “Saturation”[tiab] OR “Severe weather”[MeSH] OR “Severe weather”[tiab] OR “Snow”[MeSH] OR “Snow”[tiab] OR “Sunlight”[MeSH] OR “Sunlight”[tiab] OR “Temperature”[MeSH] OR “Temperature”[tiab] OR “Temperatures”[MeSH] OR “Temperatures”[tiab] OR “Ultraviolet rays”[MeSH] OR “Ultraviolet rays”[tiab] OR “UV index”[MeSH] OR “UV index”[tiab] OR “UV rays”[MeSH] OR “UV rays”[tiab] OR “Visibility”[MeSH] OR “Visibility”[tiab] OR “Weather”[MeSH] OR “Weather”[tiab] OR “Wind”[MeSH] OR “Wind”[tiab] OR “Wind chill”[MeSH] OR “Wind chill”[tiab] OR “Windy”[MeSH] OR “Windy”[tiab]))	
**Limiters: (“2006/01/01”[Date - Publication]**: **“2020/10/31”[Date - Publication]) AND (English[Language])**	
**Filters:** Humans	

## Appendix B

**Table 6:** Search Methods | CINAHL

**Search terms:** (MH “Exercise”) OR (MH “Physical Fitness”) OR (MH “Cardiorespiratory Fitness”) OR (MH “Physical Activity”) OR (MH “Recreation”) OR (MH “Running, Distance”) OR (MH “Running”) OR (MH “Walking”) OR (MH “Life Style, Sedentary”) OR (MH “Television”) OR (MH “Video Games”) OR (MH “Computers, Portable”) OR (MH “Computers, Hand-Held”) OR (MH “Screen Time”)OR TI “Exercise” OR “Physical Fitness” OR “Cardiorespiratory Fitness” OR “Physical Activity” OR “Recreation” OR “Running, Distance” OR “Running” OR “Walking” OR “Life Style, Sedentary” OR “Television” OR “Video Games” OR “Computers, Portable” OR “Computers, Hand-Held” OR “Screen Time” OR AB “Exercise” OR “Physical Fitness” OR “Cardiorespiratory Fitness” OR “Physical Activity” OR “Recreation” OR “Running, Distance” OR “Running” OR “Walking” OR “Life Style, Sedentary” OR “Television” OR “Video Games” OR “Computers, Portable” OR “Computers, Hand-Held” OR “Screen Time” AND (MH “Accelerometers”) OR (MH “Pedometers”) OR (MH “Step”) OR (MH “Fitness Trackers”) OR TI “Accelerometers” OR “Pedometers” OR “Step” OR “Fitness Trackers” OR “Wearable Electronic Devices” OR AB “Accelerometers” OR “Pedometers” OR “Step” OR “Fitness Trackers” OR “Wearable Electronic Devices” AND (MH “Weather”) OR (MH “Extreme Weather”) OR (MH “Seasons”) OR (MH “Temperature”) OR (MH “Humidity”) OR (MH “Heat”) OR (MH “Ice”) OR (MH “Ultraviolet Rays”) OR (MH “Sunlight”) OR (MH “Smog”) OR (MH “Light”) OR (MH “Lightning”) OR (MH “Atmospheric Pressure”) OR (MH “Rain”) OR (MH “Snow”) OR (MH “Atmosphere”) OR (MH “Meteorological Factors”) OR (MH “Climate”) OR TI “Weather” OR “Extreme Weather” OR “Seasons” OR “Temperature” OR “Humidity” OR “Heat” OR “Ice” OR “Ultraviolet Rays” OR “Sunlight” OR “Smog” OR “Light” OR “Lightning” OR “Atmospheric Pressure” OR “Rain” OR “Snow” OR “Atmosphere” OR “Meteorological Factors” OR “Climate” OR AB “Weather” OR “Extreme Weather” OR “Seasons” OR “Temperature” OR “Humidity” OR “Heat” OR “Ice” OR “Ultraviolet Rays” OR “Sunlight” OR “Smog” OR “Light” OR “Lightning” OR “Atmospheric Pressure” OR “Rain” OR “Snow” OR “Atmosphere” OR “Meteorological Factors” OR “Climate”	
**Limiters: (“2006/01/01”[Date - Publication]: “2020/10/31”[Date - Publication]) AND (English[Language])**	
**Filters:** Humans	

## Appendix C

**Table 7:** Search Methods | SPORTDiscus

**Search terms:** (DE “EXERCISE”) OR (DE “PHYSICAL fitness”) OR (DE “PHYSICAL activity”) OR (DE “RECREATION”) OR (DE “RUNNING”) OR (DE “WALKING”) OR (DE “SEDENTARY behavior”) OR (DE “SUBSCRIPTION television”) OR (DE “VIDEO games”) OR (DE “COMPUTER games”) OR (DE “SEDENTARY people”) OR TI (“EXERCISE”) OR (“PHYSICAL fitness”) OR (“PHYSICAL activity”) OR (“RECREATION”) OR (“RUNNING”) OR (“WALKING”) OR (“SEDENTARY behavior”) OR (“SUBSCRIPTION television”) OR (“VIDEO games”) OR (“COMPUTER games”) OR (“SEDENTARY people”) OR AB (“EXERCISE”) OR (“PHYSICAL fitness”) OR (“PHYSICAL activity”) OR (“RECREATION”) OR (“RUNNING”) OR (“WALKING”) OR (“SEDENTARY behavior”) OR (“SUBSCRIPTION television”) OR (“VIDEO games”) OR (“COMPUTER games”) OR (“SEDENTARY people”) AND (DE “PEDOMETERS”) OR (DE “ACCELEROMETERS”) OR (“PEDOMETERS”) OR (“ACCELEROMETERS”) OR (“STEP”) OR (“FITNESS TRACKERS”) OR (“WEARABLE ELECTRONIC DEVICES”) OR AB (“PEDOMETERS”) OR (“ACCELEROMETERS”) OR (“STEP”) OR (“FITNESS TRACKERS”) OR (“WEARABLE ELECTRONIC DEVICES”) AND (DE “WEATHER”) OR (DE “TEMPERATURE”) OR (DE “HUMIDITY”) OR (DE “ICE”) OR (DE “ULTRAVIOLET radiation”) OR (DE “SUNSHINE”) OR (DE “ATMOSPHERIC pressure”) OR (DE “SNOW”) OR (DE “COLD (Temperature)”) OR TI (“WEATHER”) OR (“TEMPERATURE”) OR (“HUMIDITY”) OR (“ICE”) OR (“ULTRAVIOLET radiation”) OR (“SUNSHINE”) OR (“ATMOSPHERIC pressure”) OR (“SNOW”) OR (“COLD (Temperature)”) OR (“ATMOSPHERE”) OR (“METEOROLOGICAL FACTORS”) OR (“EXTREME WEATHER”) OR (“CLIMATE”) OR AB (“WEATHER”) OR (“TEMPERATURE”) OR (“HUMIDITY”) OR (“ICE”) OR (“ULTRAVIOLET radiation”) OR (“SUNSHINE”) OR (“ATMOSPHERIC pressure”) OR (“SNOW”) OR (“COLD (Temperature)”) OR (“ATMOSPHERE”) OR (“METEOROLOGICAL FACTORS”) OR (“EXTREME WEATHER”) OR (“CLIMATE”)	
**Limiters: (“2006/01/01”[Date - Publication]: “2020/10/31”[Date - Publication]) AND (English[Language])**	
**Filters:** Humans	

## Appendix D

**Table 8:** Glossary of Weather Indices

Index	Definition
Temperature	Degree or intensity of heat in the environment
Precipitation	Product of condensation from atmospheric water vapor (typically rain and sleet in this review)
Wind Speed	Atmospheric quantity resulting from air traveling from high to low pressure
Photoperiod	Day length, or the period of daily illumination
Snow	Small, frozen white ice crystals generated from atmospheric water vapor at temperatures below the freezing point
Cloud Coverage	The fraction of the sky obscured by clouds in a particular location
Humidity	Concentration of atmospheric water vapor present in the environment
Visibility	Distance that can be seen due to light and weather conditions
Barometric Pressure	Pressure within the atmosphere
Windchill	Quantity of air temperature that is effectively lowered due to wind
Air Quality	A measure of how clean or polluted the air is

*Note.* Definitions obtained from the National Weather Service and the Oxford English Dictionary [150, 151]
