# Supplementary material for: Seasons, weather, and device-measured movement behaviors: a scoping review from 2006 to 2020
Source: Int J Behav Nutr Phys Act. 2021 Feb 4;18:24. doi: 10.1186/s12966-021-01091-1 (PMC7863471; doi:10.1186/s12966-021-01091-1)
Supplement: Supplementary file 1 — Additional file 1. [file 12966_2021_1091_MOESM1_ESM.docx]

*Coding Guide for Data Extraction*

| **Code** | **Guidelines** |
| --- | --- |
| Pre-screening Eligibility | Exclude if: studies are not published in English (1), studies involve animals and humans (2), correlational or association data not provided (3) only self-reported measures were used to examine physical activity or sedentary behavior (4) Qualitative study (5)  Otherwise, the paper should be included and move to quality assessment  If eligible, mark (0) for Yes; otherwise, provide the reason for exclusion |
| Study Population | Describe sample (e.g., COPD patients, elementary school kids) |
| Seasonality vs. Weather Indices | Seasonality – Results are organized by comparing seasons such as PA in winter vs. summer, but do not talk about weather indices (0)  Weather Indices – Results compare PA or SB by weather indices such as temperature, precipitation, etc (1)  Results talked about both seasonality and weather indices (2) |
|  |  |
| Country/ Region Studied | Write the country where the data was collected. Also describe region (e.g., Western US, Pennsylvania) |
| Sample Size | Mark the total number of participants that participated in the study following consent. Do not include dropouts. Include only those that were included in the results.  If this information is not provided, mark NA. |
| Age | Provide the listed descriptives (mean, st.dev, range, median).  If this information is not provided, mark NA. |
| Racial Composition | Provide the percentage of participants that fall into each listed race category.  If this information is not provided, mark NA. |
| Hispanic or Latino | Provide the percentage of participants that fall into either non-Hispanic or Hispanic categories.  If this information is not provided, mark NA. |
| Gender | Provide the percentage of participants that are female.  If this information is not provided, mark NA. |
| Education Level | If applicable, provide the percentage of participants that fall into each school grade. You may also make an educated guess based on mean age and put ‘high school’ or ‘college’.  If this information is not provided, mark NA. |
| Study Design | Mark if the study design is cross-sectional, RCT, longitudinal, etc |
| Monitoring Period | Describe the monitoring period for the study, such as two weeks, over a whole season, etc. |
|  |  |
| PA Time Frame | Were participants asked to recount their PA over the past week, month, year?  Past week = 1  Past month = 2  Past year = 3  Mixed = 4  NA = 0 |
| PA – Self report or device? | Was PA measured using self-report questionnaires or devices?  Devices = 1  Both = 2  NA = 0 |
| PA Volume | Was PA volume assessed?  Yes = 1  No = 0 |
| PA Intensity | Was PA intensity assessed?  Yes = 1  No = 0 |
| PA Duration | Was PA duration assessed?  Yes = 1  No = 0 |
| PA Frequency | Was PA frequency assessed?  Yes = 1  No = 0 |
| PA Type | Was PA type assessed?  No = 0  Aerobic = 1  Resistance = 2  Mixed = 3 |
| PA Measure | What were the specific PA measures that were used? List the specific name of the device/accelerometer and/or self-report questionnaire. |
| Wear Location | Describe where the accelerometer was worn (e.g., hip, wrist, smart phone) |
| SB Flag | Was SB assessed?  Yes= 1  No= 0 |
| SB Time Frame | Were participants asked to recount their SB over the past week, month, year?  Past week = 1  Past month = 2  Past year = 3  Mixed = 4  NA = 0 |
| SB – Self report or device? | Was SB measured using self-report questionnaires or devices?  Devices = 1  Both = 2  NA = 0 |
| SB Total Sitting Time | Was SB assessed using total sitting time?  Yes = 1  No = 0 |
| SB Context Specific | Was SB assessed using context specific questions?  Yes = 1  No = 0 |
| Which Context | Describe what context SB was measured (e.g., screen time) |
| SB Measure | What were the specific SB measures that were used? List the specific name of the device/accelerometer and/or self-report questionnaire. |
| Wear Location | Describe where the accelerometer was worn (e.g., hip, wrist, smart phone) |
| Weather Indices | List the weather indices that were reported/analyzed (e.g., precipitation, temperature, humidity, etc) |
| Weather Time Frame | Was weather measured or recorded in the past week, month, year?  Past week = 1  Past month = 2  Past year = 3  Over one year = 4  Mixed = 5  NA = 0 |
| Weather Measure | What were the specific weather measures that were used? |
| Source of Weather Data | Describe the source used to collect weather-related data |
| In addition, you will also extract specific results from the papers.  These include means, SDs and ranges for PA, SB and weather indices, if applicable.  Finally, you will extract correlational data between PA or SB and weather or seasons, if provided. This can include r correlations, beta values and/or odds ratios. | |
| PA Correlation with Weather Indices |  |
| P-Value |  |
| PA Correlation with Season |  |
| P-Value |  |
| SB Correlation with Weather Indices |  |
| P-Value |  |
| SB Correlation with Seasons |  |
| P-Value |  |
| Beta Value for PA and Weather Indices |  |
| P-value |  |
| Beta Value for PA and Seasons |  |
| P-Value |  |
| Beta Value for SB and Weather Indices |  |
| P-Value |  |
| Beta Value for SB and Seasons |  |
| P-Value |  |
| Odds Ratio (Adjusted or non) for PA and Weather Indices |  |
| P-Value |  |
| Odds Ratio (Adjusted or non) for PA and Seasons |  |
| P-Value |  |
| Odds Ratio (Adjusted or non) for SB and Weather Indices |  |
| P-Value |  |
| Odds Ratio (Adjusted or non) for SB and Seasons |  |
| P-Value |  |
| Summarize Main Findings | In 1-2 sentences please describe the main findings from the article. |
| Notes | Please list any notes that you want to share with the rest of the coders. |
